# Editorial: Resilience to stress-related mood disorders: Involvement of oxidative stress

**DOI:** 10.3389/fnbeh.2023.1137682

**Published:** 2023-01-30

**Authors:** Ana Paula Pesarico, Pietro Maria Chagas, Suzan Gonçalves Rosa, Josiane Budni

**Affiliations:** ^1^Universidade Federal do Pampa, Uruguaiana, Brazil; ^2^Faculdade da Serra Gaúcha, Caxias do Sul, Brazil; ^3^Universidade do Extremo Sul Catarinense, Criciúma, Brazil

**Keywords:** stress, depression, mice, resilient, susceptible

It is inevitable that everyone experiences stressful life events, and in some cases, acute or chronic stress can lead to depression or other mental disorders. However, most people are resilient to stress situations. Resilience refers to the ability to handle stress, trauma, or chronic adversity successfully. The resilience of an individual is thus demonstrated by their ability to adapt to psychological and physiological stress (Charney, 2004; Southwick et al., 2005; Krishnan et al., 2007). The oxidative stress, a relevant pathway in the development of mood disorders, is the result of imbalance between the production of reactive species (RS) and the organism's ability to inhibit or repair the damage caused by them through its endogenous antioxidant defenses or the use of exogenous antioxidants (Halliwell, 2011). Then, the question of this topic is “*Could oxidative stress be involved in the resilience of depression?*”

The first article published in this Research Topic by Marcololongo-Pereira et al. and cols is a review that discusses different mechanisms involved in resilience and vulnerability to depression caused by stress. The review cites the individual variability in the way animals and humans respond to stress depends on several mechanisms, such as oxidative stress, neuronal plasticity, immunology and genetic factors. The understanding of the mechanisms of resilience to stress helps in the development of new therapeutic approaches for treating or improving the quality of life of depressed patients.

The interesting study developed by Csabai et al. demonstrated that chronic mild stress exposure causes anhedonic phenotype in some animals (38%), while other animals are resilient to stress (18%). Anhedonic rats exhibited reduced mitochondrial numbers in their infralimbic cortex compared to the control group. Mitochondrial impairment can accelerate the production of RS, which further modifies biological macromolecules and alters several cellular functions (Du et al., 2009). The results of the current article provide no information on the functional consequences in the neurons, consequently, further studies are necessary to confirm the concept that the stress-induced mitochondrial damage can sensitize neurons.

Acupuncture has been practiced for thousands of years and is often used as a complementary and alternative treatment for depression (Smith et al., 2018). In the paper “*Acupuncture relieves stress-induced depressive behavior by reducing oxidative stress and neuroapoptosis in rats*,” the authors demonstrated that acupuncture present antidepressant-like effects by reducing oxidative stress products via regulating the nuclear transcription factor erythroid-2-like factor 2 (Nrf2)/heme oxygenase-1 (HO-1) signaling pathway and, consequently, prevented neuronal apoptosis in the chronic unpredictable mild stress (CUMS) model (Cheng et al.). This pathway is an endogenous defense strategy to eliminate damages caused by excessive RS production. The Nrf2/antioxidant response element (ARE) signaling is one of the critical antioxidant systems involved in the maintenance of the redox state for the defense of intracellular oxidative stress and. One of the ARE-regulated phase II detoxifying enzymes regulated by Nrf2 is HO-1, which catalyzes the degradation of heme to biliverdin, carbon oxide, and iron. In particular, HO-1 has the most abundant AREs in the promotion of genes regulated by Nrf2, and has been reported to be very important in preventing disease caused by oxidative stress, including mood diseases (Surh et al., 2009; Loboda et al., 2016; Hashimoto, 2018).

Another traditional Chinese medicine that presents a significant impact on the central nervous system, including antidepressant effects is the musk (*Moschus moschiferus*) (Sytniczuk et al., 2018). The study developed by Almohaimeed et al. showed that musk inhalation decreased oxidative stress markers in mice exposed to chronic unpredictable mild stress. Furthermore, the compound reduces the corticosterone level, inflammatory cytokines and exerts antidepressant effect in chronic unpredictable mild stress-exposed mice. These changes in behavior and biochemistry could be different pathways through which musk promotes resilience in animals, although the authors do not mention resilience.

The last article of this editorial discussed the *Sex differences in the sustained effects of ketamine on resilience to chronic stress*, which is very interesting (Okine et al.). The ketamine, a nonselective NMDA receptor antagonist, has been shown to have rapid and sustained antidepressant actions (Berman et al., 2000; Zarate et al., 2006). Furthermore, the main question is understanding why female subjects have a significantly higher risk for mood disorders than males. The present study showed that the prophylactic use of low doses of ketamine promotes resilience to some behavioral changes in the forced swimming test induced by chronic stress exposure in male mice, but fails to confer such protection in females. The results also show that ketamine has sustained effects on the expression of genes that regulate glutamatergic and GABAergic transmission in the prefrontal cortex, a region particularly vulnerable to stress, and that these effects differ in males and females. While a causal relationship between gene expression changes and behavioral resilience to stress was not established here, the results support the idea that the state of the glutamatergic/GABAergic circuits in the prefrontal cortex before exposure to chronic unpredictable mild stress might directly contribute to resilience to stress-related mood disorders. Thus, further studying sex differences in ketamine effects on those circuits might help our understanding of the mechanisms underlying increased vulnerability to emotional deregulations in females.

This Research Topic collects a number of contributions, such as chronic effects of stress, the role of oxidative stress in resilience to stress and depression, and the relationship between stress resilience and antidepressant treatments. This topic contributes to the investigation of strategies to treat depression induced by stress (Figure 1).

**Figure 1:**
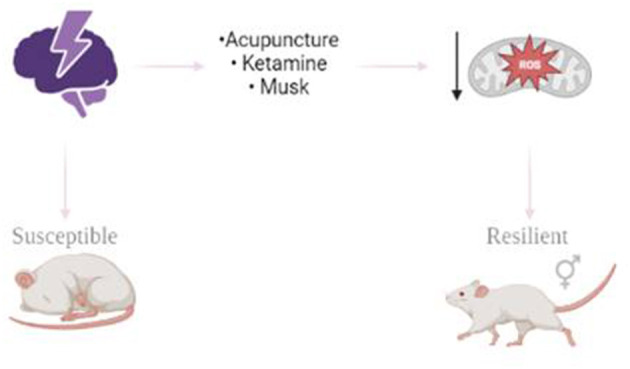
Resilience to stress-related mood disorders.

## Author contributions

All authors listed have made a substantial, direct, and intellectual contribution to the work and approved it for publication.
